# Innovative Disease Model: Zebrafish as an In Vivo Platform for Intestinal Disorder and Tumors

**DOI:** 10.3390/biomedicines5040058

**Published:** 2017-09-29

**Authors:** Jeng-Wei Lu, Yi-Jung Ho, Shih-Ci Ciou, Zhiyuan Gong

**Affiliations:** 1 Department of Biological Sciences, National University of Singapore, 14 Science Drive 4, Singapore 117543, Singapore; 2 School of Pharmacy, National Defense Medical Center, No. 161, Section 6, Minquan East Road, Taipei 114, Taiwan; ejho@mail.ndmctsgh.edu.tw; 3 Department of Clinical Laboratory Sciences and Medical Biotechnology, National Taiwan University, No. 1 Chang-Te Street, Taipei 100, Taiwan; scciou@ntu.edu.tw

**Keywords:** colorectal cancer, intestinal disorder, intestinal tumors, zebrafish

## Abstract

Colorectal cancer (CRC) is one of the world’s most common cancers and is the second leading cause of cancer deaths, causing more than 50,000 estimated deaths each year. Several risk factors are highly associated with CRC, including being overweight, eating a diet high in red meat and over-processed meat, having a history of inflammatory bowel disease, and smoking. Previous zebrafish studies have demonstrated that multiple oncogenes and tumor suppressor genes can be regulated through genetic or epigenetic alterations. Zebrafish research has also revealed that the activation of carcinogenesis-associated signal pathways plays an important role in CRC. The biology of cancer, intestinal disorders caused by carcinogens, and the morphological patterns of tumors have been found to be highly similar between zebrafish and humans. Therefore, the zebrafish has become an important animal model for translational medical research. Several zebrafish models have been developed to elucidate the characteristics of gastrointestinal diseases. This review article focuses on zebrafish models that have been used to study human intestinal disorders and tumors, including models involving mutant and transgenic fish. We also report on xenograft models and chemically-induced enterocolitis. This review demonstrates that excellent zebrafish models can provide novel insights into the pathogenesis of gastrointestinal diseases and help facilitate the evaluation of novel anti-tumor drugs.

## 1. Introduction

Colorectal cancer (CRC) is one of the world’s most common cancers. It is also the second-leading cause of cancer deaths in the United States, responsible for more than 50,000 estimated deaths in the world [1]. Although the five-year survival rate for localized CRC is >90%, most CRC patients are asymptomatic; therefore, only 40% of cases are detected at this stage. At the metastatic stage, CRC survival rate falls to 8–12%. There are five stages of CRC: aberrant foci, small adenoma/adenomatous polyps, large adenoma, adenocarcinoma, and invasion/metastasis. Risk factors for CRC include age (>50 years), being overweight, a diet that is high in red and over-processed meat, a history of inflammatory bowel disease, and smoking [1]. In addition, 5–10% of familial CRC cases include mutations in the tumor suppressor gene *APC*, and environmental risk factors have been linked to somatic mutations that can cause CRC. Non-familial cases of CRC are often related to overactivity of epidermal growth factor receptor (*EGFR*), loss of function at *APC*, and activating or allele mutations in *K-RAS*, *N-RAS*, *BRAF*, *PIK3CA*, *WNT*, and *TP53* genes. It has been hypothesized that the regulation of different CRC phenotypes could be associated with a balance between anti-proliferation, maintaining genomic stability, and oncogenes. For example, microsatellite instability results in the loss of DNA mismatch repair function, which in turn leads to overactivity of COX-2, EGFR, and/or WNT pathways and results in small adenomas. K-RAS and/or PIK3CA pathway overactivity results in large adenomas; and inactivation of the tumor suppressor gene *TP53* and downregulation of TGF-β signaling results in invasive/metastatic carcinomas [2,3,4]. In non-hereditary sporadic CRC, *APC* is mutated in 85% of cases, *TP53* is mutated in 40–50% of cases, *PIK3CA* is mutated in 35% of cases, and *TGFBR2* is mutated in 45–50% of cases [2].

*APC* mutations activate the WNT pathway by increasing the amount of β-catenin that is translocated into the nucleus and enhancing the transcription of various oncogenes [5]. Causes of *APC* inactivation include hypermethylation of the *APC* promoter, germline mutations, and somatic mutations [6]. The *APC* gene is responsible for approximately 75% of mutations or the loss of heterozygosity (LOH) in CRC. Most *APC* mutations are clustered in the mutation cluster region that is located between codons 1282 and 1581 [7]. Previous studies have demonstrated the effects of *APC* restoration in cancerous mice, whereby tumor cells were replaced by normal cells, and some evidence suggests that *APC* restoration therapy could have similar benefits for humans [8]. Another study showed that, in a CRC cell line, β-catenin activates a set of 162 target genes associated with the WNT pathway; however, no conclusions could be drawn regarding the effect of these genes on cancer prognosis [9].

The first proposed model of genetic events that led to the development of CRC involved point mutations in *K-RAS* [10]. Specifically, point mutations in codons 12, 13, and 61 of *K-RAS* activate an enzyme that increases RAS signaling. Indeed, *K-RAS* has been found to be mutated in 30–40% of CRC cases, and in 60–90% of hyperplastic or non-dysplastic aberrant crypt foci [11,12]. Mutations in codon 12 of *K-RAS* are associated with more advanced tumor stages [13], and RAS signaling further activates the Raf-MEK-ERK pathway, the PI3K/AKT/PKB pathway, and Ral small GTPases [14]. Furthermore, most human CRC cases that involve PI3K gene mutations also involve *K-RAS* mutations [15]. *AKT1* and *AKT2* genes enhance tumor growth by promoting epithelial-to-mesenchymal transition (EMT) through PI3K activation [16,17], and the tumor suppressor gene of the *PTEN* antagonist PI3K/AKT pathway induces AKT-regulated tumor metastasis through loss-of-function mutations [18]. Finally, the MEK/ERK and PI3K/AKT pathways often converge to activate a cap-dependent translation, which can inhibit metastasis through the knockdown of survivin [19].

In the majority of human tumors, TP53is dysfunctional. Several tumors display a gain of function mutation in TP53, which results in mutated TP53 (mutTP53) proteins [20]. MDM2 can also bind and inactivate the mutTP53 isoform. In CRC tissues, the overexpression of protein and mRNA was observed in the spliced isoform MDM2-B. Protein and mRNA overexpression is mainly associated with the mutTP53 protein as MDM2-B binds to MDM2, which in turn allows mutTP53 to accumulate in cells [21]. Indeed, a previous report showed that a loss-of-function mutation in TP53 affects 44.9% of colorectal adenoma cases, 42.22% of single primary CRC cases, and 43.75% of multiple primary CRC cases [22].

In current clinical practice, the only option to treat unresectable metastatic CRC is conventional chemotherapy. Although chemotherapy tends to relieve initial symptoms, resistance generally develops within six months. Life-extending agents, such as conventional cytotoxics and targeted therapeutics, are frequently used, including 5-fluoracil, capecitabine and topotecan, bevacizumab, cetuximab, and panitumumab [23,24]. Unfortunately, CRC patients who receive these agents also develop a resistance to them, and have a final average survival rate of only 13.3 months [25]. So far, zebrafish has been well used to study several intestinal cancer and disorder. Here we give a broad view to understand how zebrafish involved in those studies.

## 2. Development and Anatomy of the Gastrointestinal Tract in Zebrafish

The digestive system plays a critical role in vertebrate physiology and the intestinal anatomy among amniotes is highly conserved. The zebrafish is a powerful animal model in the study of intestinal development. Moreover, genes and organ functions are also well conserved between zebrafish and higher vertebrates [26,27,28]. For example, in zebrafish, the internal lining of the intestines forms a ridge (and not villi). This unique characteristic can be observed in cross-sections of mammalian intestines as well. Although zebrafish lack a stomach, crypts, Paneth cells, submucosal glands, and the organization of lymphoid structures, the zebrafish intestine is a simple but unique organ in vertebrate intestinal biology.

The morphological development of zebrafish intestine has been comparatively well studied in embryos and larvae [29,30,31,32]. However, zebrafish lack a morphologically and functionally distinct stomach, and do not express genes that encode precise gastric functions. Zebrafish do have an intestinal bulb with a lumen that is larger than the posterior part of the intestine. This intestinal bulb may function as a container that is comparable to the stomach. The digestive enzymes and solute transporters are present in the anterior and mid intestines [33]. A previous report indicated that because the intestinal bulb of zebrafish lacks gastric glands, the pH in the zebrafish intestines never falls below 7.5 [34].

Early research revealed that the zebrafish digestive tract develops in a segmental fashion, and that development begins during the mid-somite stages. Gut tube formation begins during mid- to late-somite stages (~18 somites) in zebrafish; however, in mammals, the gut begins to form during the early-somite stages (1–2 somites). At the 18-somite stage, a continuous thin layer of endoderm becomes distinguishable, which will eventually give rise to the primitive gut endoderm. Although gut formation occurs later in zebrafish, the temporal progression of gut tube formation is similar to that of mammals: the rostral gut of zebrafish develops first, followed by the hindgut and midgut [29].

Zebrafish gut development begins at almost 20 h post fertilization (hpf) and proceeds as follows. Firstly, endodermal precursors form the primitive gut: a thin, rod-like cell layer that lengthens from the future mouth to the future anus of the embryo. Progenitor gut cells then polarize to become columnar epithelium, and junctional complexes form between cells, which are required for lumen inflation and the establishment of the epithelial barrier. These developmental progressions occur in conjunction with cell proliferation along the total length of the intestinal tube. Proliferation is downregulated at approximately 72 hpf, at which time the intestinal epithelial cells are differentiated into the three lineages of mature gut epithelium, including absorptive enterocytes, mucus-producing goblet cells, and hormone-secreting enteroendocrine cells. Around 120 hpf, the yolk is entirely absorbed, and gut development is almost complete. At this time, the embryo is able to feed and digest. Previous studies have described the zebrafish intestine as a tapered tube that begins at the esophageal junction and is folded into three segments: (1), the large diameter rostral intestinal bulb, which is characterized by an expanded lumen and epithelial folding, and is primarily comprised of enterocytes and enteroendocrine cells; (2), the mid-intestine, which is demarcated by the presence of goblet cells and enterocytes with large, supranuclear vacuoles; and (3), the small diameter posterior intestine, which does not possess endocrine and goblet cells. Even though zebrafish do not have five intestinal segments like mammals (i.e., jejunum, duodenum, cecum, ileum, and colon), zebrafish and mammalian intestines do share functional homology. For example, in both zebrafish and mammals, growth factor gradient combinations of bone morphogenetic protein (Bmp), fibroblast growth factor (Fgf), and wingless-type MMTV integration site (Wnt) at the posterior end of the endoderm are able to regulate intestinal development. Additionally, retinoic acid (RA) signaling plays a dose-dependent role in patterning the anterior–posterior (A-P) body axis, including the endoderm [31,32].

Wang et al. [28] previously performed a microarray analysis of adult zebrafish guts, in which guts were separated into seven sections of equal lengths (from anterior 1 to posterior 7). Using metabolic gene data, they were able to confirm the presence of three distinct gut regions. Furthermore, segments 1 to 5 (*S1* to *S5*) showed high expression of intestinal markers that are conserved in humans and mice: fatty acid binding protein 2 (*fabp2*), villin 1 (*vil1*), and apolipoproteins 1 and 4 (*apoa1* and *apoa4*). *Fabp2, Apo1*, and *Apo4* all participate in lipid metabolism. Wang et al. [28] also reported that the *Vil1* gene plays a role in regulating anti-apoptosis in small intestinal epithelial cells. Additionally, aquaporin 3 (*aqp3*) and cofilin1 (*cfl1*) (which are biomarkers of the large intestine in mammals) were found to distinguish genes associated with sections *S1* to *S4* from genes associated with *S5* to *S7* in zebrafish. Furthermore, *Cfl1* was reported to regulate the dynamic stabilization of *actin* filaments, and *aqp3* was found to participate in water absorption. Wang et al. [28] also indicated that *S1* to *S5* share molecular characteristics with the small intestine of mammals, and that *S6* and *S7* share similar characteristics with the large intestine of mammals. Finally, *S5* was found to form a transition segment, and was surmised to be the dorsal fraction of the mid-gut that participates in mucosal immunity [28]. However, in order to further investigate the correlation of these genes between diseases, to develop the transgenic zebrafish is quite critical.

## 3. Developments in Transgenic Zebrafish Technology that Enable the Exploration of Intestinal Tumors Using a Constitutive or Inducible Expression System

Significant progress has been made in the development of transgenic technology, which is an essential technique that is used in research and employed in a variety of model organisms [35,36]. A variety of transgenic expression systems exist, including constitutive and inducible systems [37]. Traditionally, establishing a transgenic fish model involved injecting fish embryos with (1) artificial chromosomes of recombinant bacteria [38], (2) supercoiled [39] or linear DNA [36], and (3) linearized ISce-I meganuclease [40]. Recently, however, the use of Sleeping Beauty (SB) [41], Ac/Ds [42], and Tol2 [43] transposon-based systems has effectively increased transgenic efficiency in zebrafish. The development of SB was based on DNA sequences of Tc1-like elements (TcEs) from teleost fish species [44,45]. When the SB transposon plasmid and SB transposase mRNA are co-injected into fertilized eggs, the SB transposon vector is transposed from the plasmid to the zebrafish genome, and the transposon insertions are transmitted to the next generation of zebrafish germline [46]. The Tol2 transposon element was found in the medaka genome, which can be efficiently excised and integrated into the zebrafish genome. This enables transgenic lines to be generated by co-injecting fish with Tol2 mRNA and vector plasmid [43]. In a plant transposon system, the maize Dissociation (Ds) element is capable of effective Activator (Ac) transposase-mediated transposition in the zebrafish, yielding high transposition frequencies and efficient germline transmission rates [42].

Previous studies noted that the overexpression of oncogenes can lead to serious tumors, early embryonic developmental abnormalities, and death, which prevents oncogene effects from being comprehensively characterized. Controlling gene expression through the use of an induction system can help address this problem. Currently, widely used induction systems include heat shock, Cre-loxP, GAL4-UAS, Tet-On, Tet-Off, and mifepristone. These systems regulate the duration and dosage to achieve spatiotemporal control of transgene expression in fish during both the embryonic and adult stages [37,47].

The important role of intestinal-type fatty acid binding protein (I-FABP; also known as FABP2) has been observed in vertebrates. Specifically, this role involves the intracellular binding and trafficking of long chain fatty acids. Mammalian gene promoters and ubiquitous or endogenous tissue-specific promoters are able to drive the expression of green fluorescent protein (GFP) and red fluorescent protein (RFP) transgenes in zebrafish [48,49]. Zebrafish 4.5-kb *FABP2* gene promoter also drives intestine-specific GFP/RFP expression in the zebrafish. Indeed, previous research noted the ability of the *FABP2* gene promoter to direct GFP/RFP fluorescent expression in the intestinal tube from three days post-fertilization (dpf) (when zebrafish were in the larval stage) until the adult stage [49,50]. The first transgenic fish model of an intestinal tumor was developed through the expression of the *H. pylori* virulence factor *cagA*, which was in turn controlled by the 1.6-kb *FABP2* promoter in *tp53* mutant background zebrafish (tp53^M214K^) [51]. Heat shock-inducible Cre/Lox expression controlled by the *β-actin* promoter of human *K-RAS^G12D^* has also been induced in intestinal epithelial tumors alongside several other tumors in zebrafish [52].

## 4. Zebrafish Models for Intestinal Tumors and Disorders

The zebrafish is a powerful animal model that can be used for the forward and reverse genetic analysis of vertebrate embryogenesis, organ development, disease, tumors, and toxicology. Many zebrafish mutants or transgenic lines have also proven to be excellent animal models for a variety of human diseases and tumors [53]. Experimental carcinogenesis studies have illustrated the development of tumors that can occur in the wild in virtually all organs in zebrafish [54,55]. Furthermore, the histopathology of intestinal neoplasia in zebrafish is similar to the histopathology of intestinal neoplasia in humans [56,57]. One study found that the histological signs of cancer in zebrafish included preneoplastic intestinal changes, such as hyperplasia, dysplasia, adenocarcinoma, small cell carcinoma/carcinoid-like, tubular/tubulovillous adenoma, and enteritis [58]. According to histological diagnosis, the intestinal carcinomas were comprised of neuroendocrine cells. Immunohistochemistry analysis of cytokeratins (that used human epithelial (cytokeratin wide spectrum screening (WSS), AE1/AE3) or neuroendocrines (S100, chromogranin A) markers) confirmed that the majority of intestinal tumors in a cohort of zebrafish were carcinomas [59].

Mutations in the *APC* gene have been identified as being responsible for human familial adenomatous polyposis (FAP) syndrome [60,61]. *APC* gene mutations can also lead to the development of multiple colorectal adenomas following somatic inactivation of the remaining allele via carriers of germline truncating mutation [62]. Similarly, results from a mouse model revealed that multiple tumors developed in the small intestine when the *APC* gene contained a heterozygous truncating mutation [63]. Finally, *APC* was also found to be a key inhibitory gene in the WNT/β-catenin pathway [64].

Zebrafish *apc*-mutants carry a premature stop codon in the putative mutation cluster region (MCR) of *APC*, which mimics the mutations found in FAP patients. Homozygous *apc*-mutant fish embryos die between 72 and 96 hpf. The unusual features that characterize these fish include an aberrantly developed gut, liver, and pancreas. [65]. In one study, 17.6% of adult *apc*-mutant fish (aged >15 months) developed spontaneous liver tumors, and 11.8% of these fish developed spontaneous intestinal tumors. The *apc*-mutant fish appear to have polyps, which is a mammalian resemblance. These intestinal disturbances were observed in the disorganized large structures with ramifications of the villi, which are frequently embedded in fibrovascular stroma. The pathologic lesions were classified as adenomatous polyps. These lesions showed pseudostratification of nuclei, loss of goblet cells, and a high N/C (nuclear-to-cytoplasmic) ratio that is consistent with dysplastic epithelium. In intestinal adenoma tissue of *apc*-mutant fish, high levels of β-catenin accumulated in proliferating cells of both the cytoplasm and nucleus. In addition, 58.3% of *apc*-mutant fish (aged 14 months) treated with 7,12-dimethylbenz[a]anthracene (DMBA) showed intestinal adenomas, whereas only 20.5% of wild-type fish treated with DMBA showed intestinal adenomas. This well-established *apc*-mutant fish is a bona fide tumor suppressor, similar to its mammalian counterpart. Furthermore, the loss of heterozygous *apc*-mutant fish resembles the cancer phenotype in mammals [66]. Recent studies have also used zebrafish to investigate the genetic relationship between mitochondrial pyruvate carrier 1 (*MPC1*) and *APC*. Data from this research has demonstrated that (1) *apc* controls the levels of *mpc1* and (2) the knockdown of *mpc1* recapitulates the phenotypes of impaired *apc* function, such as failed intestinal differentiation. Moreover, exogenous human *MPC1* RNA rescued failed intestinal differentiation in apc-deficient zebrafish [67].

In recent years, zebrafish models have been developed to investigate whether the mutations of human *H-RAS*, *N-RAS*, *K-RAS* or zebrafish *k-ras* caused tumorigenesis, including models that illustrate chordoma [68], melanoma [69], rhabdomyosarcoma [70,71], brain tumors [72], gill tumors [73], liver tumors [74], pancreatic tumors [75], and other tumors that faithfully recapitulate human disease symptoms. The shock-inducible Cre/Lox-mediated *K-RAS^G12D^* transgenic fish approach can be conditionally caused within transgenic fish via heat shock treatment. This heat shock-inducible recombination approach has enabled the generation of multiple types of *K-RAS^G12D^*-induced rhabdomyosarcomas (RMS), myeloproliferative disorders, and intestinal epithelial hyperplasia. For example, *K-RAS^G12D^* activation in intestinal epithelial cells triggered intestinal hyperplasia in zebrafish. This is consistent with findings that indicated 50% of human CRC cases have *RAS* gene mutations [76]. In zebrafish, cases of intestinal epithelial hyperplasia were characterized by epithelial cells with severely disorganized intestinal epithelial architecture. Specifically, these cells showed several foci forming large outgrowths in the gastrointestinal cavity of *K-RAS^G12D^* fish. The *K-RAS^G12D^*-induced tumor and hyperplasia transgenic models generated here are similar to their related human malignancies [52].

Two *tp53* mutant (tp53^N168K^ and tp53^M214K^) fish that harbor missense mutations in the DNA-binding domain of the *tp53* gene have been mutagenized by *N*-ethyl-*N*-nitrosourea (ENU). Both of the mutated *tp53* alleles were dominant-negative, which is orthologous to cancerous mutations in human *TP53* cells [77]. Homozygous tp53^M214K^ mutant fish spontaneously formed tumors starting at the age of 8.5 months, and the tumor incidence rate was 28% when fish were 16.5 months old. The most common malignant tumors in *tp53* mutant zebrafish were peripheral nerve sheath tumors [77]. According to the literature, *tp53* zebrafish mutants have been used to study the gastrointestinal tumorigenesis of liver cancer [78] and intestinal tumors [51], as the synergistic interactions between target genes and the *tp53* mutation encourage the formation of these tumors. The allelic loss of TP53 has also been observed in human CRC, and is thought to be a late event that occurs during the transition from adenoma to carcinoma [79]. Data from mouse tumor models have also suggested that TP53 inactivation is an essential event in the progression of pancreatic cancer [80], liver cancer [81], and colorectal cancer [82]. However, assessing the role played by zebrafish *tp53* and *tp53*-related pathways in both wild-type and mutant fish could facilitate a better understanding of the role played by this pleiotropic pathway [79].

Primary risk factors of human gastrointestinal cancer include infection with *Helicobacter pylori* or other bacterial strains that carry the virulence factor *cagA*. To elucidate the mechanism that underlies the *cagA* promotion of cancer formation, the expression of *cagA* by *β-actin* or *FABP2* has been studied in both wild-type and *tp53* mutant zebrafish [77]. The expression of *cagA* led to significantly increased rates of intestinal epithelial cell proliferation in zebrafish by expressing either the wild-type or a phosphorylation-resistant form. Furthermore, the target genes of the WNT pathway, such as *cyclinD1*, *axin2*, and *myca*, were significantly upregulated. Co-expression of *cagA* with the loss-of-function allele *axin1* also increased the proliferation of intestinal cells; however, co-expression of *cagA* with *tcf4* (a null allele of the key *β-catenin* transcriptional cofactor) restored intestinal proliferation to that of wild-type fish, which showed normal intestinal architecture, with a single layer of epithelial cells lining the mucosal folds, at 18 months. Additionally, overexpression of *cagA* (under the control of ubiquitous *β-actin* or the intestine-specific *FABP2* promoter) induced mucosal fold epithelial hyperplasia, dysplasia within mucosal sulci, and mucosal fold fusion in the intestines of 12-month-old wild-type fish. Intestinal epithelial hyperplasia and definitive neoplasia, such as adenocarcinoma and small cell carcinoma, were also observed in 12-month-old *tp53* and *FABP2*-*cagA/*tp53^M214K−/−^ mutant fish. Finally, a synergistic interaction between *cagA* and the loss-of-function mutation in the tp53 allele were found to facilitate the formation of small cell carcinoma and adenocarcinoma in the intestine of *FABP2*-*cagA/*tp53^M214K−/−^ transgenic fish [51]. This model established that the intestinal tumors transgenic model would be a great advantage in the study of *cagA*-associated gastrointestinal cancers (Table 1).

## 5. The Potential of Zebrafish Xenograft Models to Benefit the Study of CRC Tumor Metastasis and Drug Screening

Pioneering work conducted by several laboratories has indicated that zebrafish embryos have the potential to benefit large-scale drug screening applications [83,84]. Zebrafish can be arrayed in a variety of isolated 12-well, 24-well, and 96-well plates (or even larger plates). For this, fish are bathed in water that contains the small molecules or chemical compounds of interest, a procedure that is ideally suited for high-throughput screening [85]. Over the past decade, the study of these zebrafish models is the most relevant research in clinical relevance [83,84]. Living cells or tissues can be transposed from one species to another using a xenograft method [86].

Casper zebrafish mutants [87] and vascular fluorescent reporter transgenic zebrafish lines (*fli1a:EGFP*) [88] have also previously been generated. Casper zebrafish lack melanocytes and iridophore cells and are therefore transparent from the embryonic stage through to adulthood; the *fli1a:EGFP* reporter line permits the visualization of both blood and lymphatic vessels. Whole-mount alkaline phosphatase vessel staining assays can be used with *fli1a:EGFP* transgenic embryos to investigate angiogenesis, tumor invasion, tumor metastasis, and anti-vascular endothelial growth factor (VEGF) drugs, as well as to disseminate cancer cells [37,89]. In addition, the lymphocyte-deficient *rag2E450fs* (casper) mutant transparent line (ZFIN allele *rag2fb101*) has been engrafted into a wide variety of normal and cancerous zebrafish cells to (1) optimize cell transplantation, (2) improve the visualization of fluorescently-labelled cancer cells at a single-cell resolution, and (3) analyze interactions between tumor cells and other key players in the tumor microenvironment. The tumor cells transplantation method using *rag2E450fs* (casper) mutant also enables cancer processes to be visualized at a single-cell resolution in vivo [90,91]. Indeed, in conjunction with advances in imaging technology, these mutant lines have created new opportunities for zebrafish xenograft models to be employed in the study of tumor cell metastasis and in the screening of novel drugs [37,92].

In another study, researchers labeled two human colorectal cancer cell lines (SW620 and SW480) with 1,1′-dioctadecyl-3,3,3′3′-tetramethylindocarbocyanine (DiI), a lipophilic fluorescent tracking dye. After labeling, cells were collected and injected into the yolk sac or perivitelline space of 2 dpf zebrafish embryos. The colorectal SW620 cells then proliferated, migrated, and formed compact cancer cells. Masses were identified seven days later near the intestinal lining [93]. Conversely, the DiI-labeled non-transfected SW480 cells were irradiated with 0–10 Gy immediately following injection. After 24 h, the number of cells that had disseminated into the tail was determined, wherein a dose-dependent relationship was observed between disseminated cells and radiation intensity (e.g., the number of cancer cells found in the tail of embryos that had received a radiation dose of 10 Gy had significant increased). SW480 cells knocked down for *AEG-1* were also injected into the zebrafish perivitelline cavity and irradiated with 0 Gy or 10 Gy. The number of disseminated cells found in the tail of fish injected with *AEG-1* knockdown cells was significantly lower than that of the control fish. Furthermore, the number of non-transfected SW480 cells (negative control) was significantly higher in the tail of irradiated embryos than in the control embryos that did not receive radiation treatment. Yet, there was not a significant increase upon radiation for the SW480 *AEG-1* knockdown cells compared with the unirradiated control. In summary, this study showed (1) that tumor invasion can be enhanced by radiation, but (2) *AEG-1* knockdown can inhibit this process. This was also the first study to demonstrate that zebrafish comprise an excellent model for the study of early events in radiation-enhanced tumor invasion [94].

In yet another study, stable fluorescent colorectal carcinoma cells expressing GFP protein (HCT-116-GFP) were injected into the yolk sac of 2 dpf embryos. These animals were maintained in 96-well plates at 35 °C for 24 h to facilitate tumor cell proliferation and embryo recovery. Different doses of crambescidine-816 (0.5, 1, and 2 µM) or 5′-fluoracile (500 µM) were then administered for 48 h. This was the first study to demonstrate that crambescidin-816 induces colorectal carcinoma in a zebrafish xenograft model [95]. Recent literature further revealed that CRC zebrafish patient-derived xenografts (zPDX) derived from surgery resected CRC samples and treated with the same treatment administered to the patient provide proof of concept experiments that compare responses to chemotherapy and biological therapies between patients and zPDX [96].

**Figure 1:**
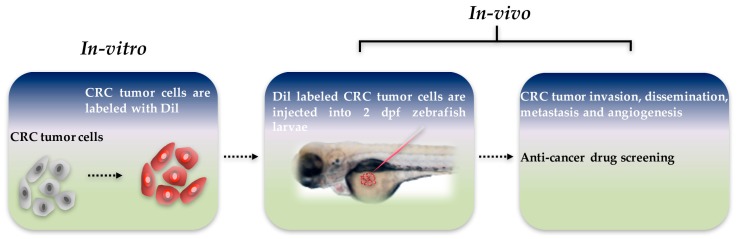
Schematic diagram of zebrafish xenograft model. Colorectal cancer (CRC) tumor cells were labeled with 1,1′-dioctadecyl-3,3,3′3′-tetramethylindocarbocyanine (DiI) dye in vitro, and approximately 300 tumor cells are injected into the yolk sac of each two days post-fertilization zebrafish larvae. Tumor invasion, dissemination, metastasis, and angiogenesis can be visualized, and anti-cancer drug screening can be conducted in vivo in a matter of days.

Zebrafish xenograft models can be used for the development and evaluation of anti-cancer drugs. These models enable the response of human tumors to potential anti-cancer drugs to be observed directly. Human tumor material is particularly targeted for primary patient-derived biopsy specimens, and is often hard to maintain in vitro. Therefore, zebrafish models represent an effective way to reduce the time and expense required to conduct cancer treatment research [92]. Additional future advances could allow zebrafish to become an excellent in vivo drug testing model, and may provide an inexpensive and highly scalable platform that can be used in preclinical trials (Figure 1) [97].

## 6. Chemically Induced Enterocolitis in Larvae and Adult Zebrafish

Inflammatory bowel disease (IBD) is a chronic inflammatory disease of the gastrointestinal tract. The symptoms of IBD include diarrhea, abdominal pain, weight loss, ulceration, perforation, and bowel obstruction. IBD is classified into two major forms, Crohn’s disease (CD) and ulcerative colitis (UC), both of which can cause high morbidity and mortality. A key feature of CD is the aggregation of macrophages. CD often forms non-caseating granulomas that affect the whole intestine. UC is characterized by an increase in neutrophils and a depletion of goblet cell mucin, and usually affects the mucosal lining of the colon and rectum. Currently, the onset and pathogenic origin of IBD remain unclear; however, zebrafish provide a platform to investigate IBD (Table 2). Specifically, chemically-induced enterocolitis is often used to investigate intestinal inflammation. A variety of zebrafish progeny are suitable for the analysis of chemically induced inflammation [98].

Chemically-induced enterocolitis models have already been established in both zebrafish larvae and adult zebrafish, which could cause intestinal epithelium damage and immune cell recruitment. The first developed an adult fish model for enterocolitis by intrarectally injecting 0.2% oxazolone, a haptenizing agent. Oxazolone caused the architecture of the intestinal wall to become thick and disrupted and also caused the intestine to lose goblet cells and undergo an influx of neutrophils and eosinophils. Furthermore, oxazolone led to an increase in the expression of cytokines, such as interleukin-1 β, tumor necrosis factor-α, and interleukin-10. That research further showed that (1) intestinal microbiota contribute to oxazolone-induced enterocolitis, and (2) vancomycin treatment led to an outgrowth of fusobacteria and reduced the percentage of proteobacteria. Indeed, zebrafish treated with vancomycin showed a reduction in oxazolone-induced enterocolitis score, decreased neutrophil infiltration, and diminished cytokine expression [99].

In research that demonstrated how a zebrafish model can be used in high-throughput chemical screening [100], Fleming et al. established a zebrafish enterocolitis model by immersing 3 dpf larvae in 75 µg/mL of 2,4,6-trinitrobenzene sulfonic acid (TNBS) (which has also been used to induce intestinal inflammation in mice). Fluorescent dye was then used to image live zebrafish larvae and analyze intestinal architecture and peristalsis in vivo. TNBS-induced enterocolitis was found to reduce villus length, enlarge crypts, decrease peristalsis, increase the number of goblet cells, and increase the expression of tumor necrosis factor-α. Moreover, TNBS was found to cause not only intestinal damage, but also skin lesions. However, a separate study by Oehlers et al. noted that larvae did not show skin lesions if they (1) were immersed in lower doses of TNBS or (2) were immersed in the 75 mg/mL dose for less than three days, to influence the analysis of enterocolitis. However, treatment with prednisolone or 5-amino salicylic acid slowed the reduction of the progression of enterocolitis. The data also showed that the number of goblet cells and proliferating cells both increased. Moreover, TNBS induced leukocytosis and reduced the branches of subintestinal vasculature, and a correlation was found between microbiota and TNBS-induced mortality. A potential explanation for this correlation is that microbiota are able to induce the transcription of pro-inflammatory cytokines, such as *il-1beta, tnf-alpha, ccl-20* and *il-8*, which promote inflammation [101].

TNBS has also been directly injected into the rectum of adult zebrafish to induce enterocolitis. Histological analysis revealed that this caused intestinal villi to become thicker and shorter, but did not affect the number of goblet cells. The survival rate of zebrafish with TNBS-induced enterocolitis was found to be related to microbiome diversity. In addition, TNBS caused pro-inflammatory and anti-inflammatory cytokines to be upregulated, and the expression of MCH and its receptor to increase. These findings suggest another potential therapeutic approach to IBD [102]. He et al. posited that TNBS may reduce the diversity of intestinal microbiota, which induces enterocolitis in larvae. Those researchers further showed that the reduction in microbiome diversity was due to an increase in Proteobacteria and a decrease in Firmicutes. It is possible that those chemicals influenced the composition of intestinal microbiota, which may have activated the TLR signaling pathways to initiate mucosal immune-mediated inflammation. However, intestinal damage and TNF-α overexpression was observed before the occurrence of microbiota dysbiosis, which suggests that a feedback loop exists between the interactions of the host and microbiota that perpetuated the inflammatory response [103]. He et al. also conducted additional experiments using germ-free fish, and found that TNBS-induced enterocolitis was not severe, even though toll-like receptor 3, MyD88, TRIF, NF-κB, and TNF-α were expressed. When microbial bacteria colonize, the characters revert to TNBS-treated conventionally-reared zebrafish. In summary, the zebrafish larvae model revealed that gut microbiota play a key role in TNBS-induced enterocolitis [104].

Dextran sodium sulfate (DSS) is a detergent that is also commonly used in animal models of IBD [105]. The highest tolerated dose (i.e., that did not induce significant mortality) had a concentration of 0.5%. Similar to enterocolitis induced by TNBS, DSS-induced enterocolitis caused liver discoloration and increased the number of neutrophils that migrated to the intestine. Moreover, DSS was found to upregulate the transcription of *ccl20*, *il1b*, *il23*, *il8*, *mmp9*, and *tnfa*. However, unlike enterocolitis induced by TNBS, DSS-induced enterocolitis led to the overgrowth of bacteria and reduced the proliferation of cells. Those researchers further observed that DSS induced the accumulation of acidic mucins in the intestinal bulb. This phenotype was associated with microbiota, but was not related to neutrophilic inflammation. Another previous study indicated that increased mucin secretion could prevent TNBS-induced enterocolitis. The mucosecretory phenotype of DSS was used to assess protection against TNBS-induced enterocolitis. DSS was found to reduce mortality and neutrophilic inflammation. In addition, retinoic acid (RA) was a conserved modulator of intestinal epithelial cell differentiation. Other evidence has also suggested that RA is able to suppress mucin secretion, and that co-treatment with RA and TNBS increases the mortality rate associated with TNBS-induced enterocolitis. Furthermore, pre-treatment with DSS in conjunction with RA may reduce the protective ability of DSS. Results of that study emphasized the importance of mucin secretion during enterocolitis progression [106]. In addition, Oehlers et al. wrote an additional technical report that focused on enterocolitis induced by TNBS and DSS that introduced several methods to assess intestinal damage and inflammatory processes. Those researchers reported that, if the proper genetic and imaging tools are employed, a zebrafish model could be useful for (1) high-throughput drug screening and (2) investigations that seek to elucidate the mechanisms that underlie drug efficacy [107]. They also found that most of the anti-inflammatory drugs they studied were able to protect against chemically-induced enterocolitis. For example, cholecystokinin (CCK) and dopamine receptor agonists were found to reduce enterocolitis-associated inflammation, thus providing a new therapeutic target [108]. TNBS and DSS were also used to establish an inflammatory lymphangiogenesis model, because they induced intestinal vascular endothelial growth factor (VEGF) receptor-dependent lymphangiogenesis in zebrafish larvae. This evidence suggests that macrophage recruitment and macrophage expression of intestinal VEGFs are correlated with intestinal inflammatory lymphangiogenesis. Therefore, this study helped to elucidate the mechanism underlying inflammatory lymphangiogenesis during IBD [109].

## 7. Concluding Comments

**Figure 2:**
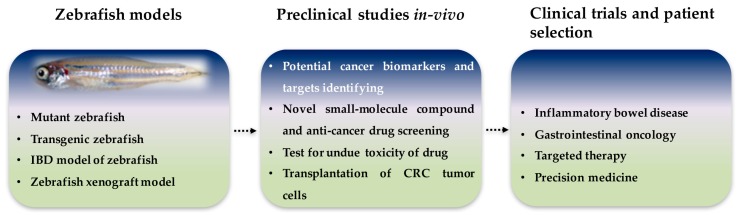
Roles of zebrafish intestinal disorder and tumor models in present and future research. Zebrafish is an ideal genetic and disease model system which is accessible for rapid screening and experimental manipulation for preclinical studies. In the future, zebrafish models could be used for patient selection in clinical trials.

In this review paper, we provided an overview of the latest research that employed zebrafish models of intestinal disorders and tumors. Zebrafish have been found to share a substantial number of conserved genes with humans, and zebrafish tissue morphology is also similar to that of humans. Recent reports have used cells or mouse models to elucidate the disease and its pivotal role in cancer initiation. The zebrafish model offers unique advantages and can greatly contribute to the field of cancer research. Therefore, the development of zebrafish models for intestinal disorders and tumors should greatly benefit studies that seek to investigate potential cancer treatments or the mechanisms that underlie tumorigenesis. At present, zebrafish models have been established for several bowel diseases and intestinal tumors, including *apc*-mutant, *K-RAS^G12D^*, *cagA*, and *cagA*/tp53^M214K−/−^. Zebrafish models have also been used to investigate preclinical and primary tumors, tumor metastasis, cancer biomarkers, targets and small molecule drugs in human digestive organs. Although some understanding of the molecular mechanisms and biological functions associated with intestinal diseases and intestinal neoplasms exists, current knowledge is limited. With the ability to facilitate high-throughput screening for the discovery of novel therapeutic agents, zebrafish could become increasingly important as an in vivo model (Figure 2). Finally, as the utility of zebrafish models in the study of cancer becomes more widely accepted it may promote further drug discovery in the future, thus one day ahead of treatment and prognosis in human patients.

## Figures and Tables

**Table 1:** Zebrafish animal models of intestinal disorder and tumors.

Gene Name	System or Mutation Site	Phenotypes	Stage	Refs.
*mpc1*	Knock down of *mpc1*	Failed intestinal differentiation	96 hpf	[67]
*apc*	Stop codon in the MCR	Liver and intestine tumors	15 months	[65]
*apc+DMBA*	Stop codon in the MCR	Intestinal adenomas	14 months	[66]
*tp53*^M214^	Point mutations in the DBM	Peripheral nerve sheath tumors, intestinal hyperplasia and adenocarcinoma	12 months	[51,77]
*K-RAS^G12D^*	*HSP*-inducible Cre/Lox	Rhabdomyosarcomas, myeloproliferative disorder and Intestinal hyperplasia	0.8–3.4 months	[52]
*cagA*	*B-actin*-constitutive expression	Intestinal hyperplasia, dysplasia and mucosal fold fusion	12 months	[52]
*cagA*^EPISA^	*B-actin*-constitutive expression	Normal	12 months	[51]
*cagA*	*FABP2*-constitutive expression	Intestinal hyperplasia, dysplasia and mucosal fold fusion	12 months	[51]
*cagA*+*tp53*^M214^	*FABP2*-constitutive expression	Intestinal hyperplasia, adenocarcinoma and small cell carcinoma	12 months	[51]

MCR: Mutation cluster region; DMBA: 7,12-dimethylbenz[a]anthracene; DBM: DNA-binding domain; *cagA*^EPISA^: A *cagA* mutant lacking ELISA motifs; *HSP*: Heat shock promoter; *FABP2*: Intestinal fatty acid-binding protein promoter.

**Table 2:** The advantages and limitations in the inflammatory bowel disease model of zebrafish.

Items	Larvae	Adult Zebrafish
**Advantages**	Many individuals	Less skin damage
	Live imaging	Adaptive immune involved
	Germ-free derivation	
	Colonization with specific bacteria	
	High-throughput drug screening	
	Easy operation	
**Limitations**	Chemically-induced skin damage	With craft
		Less sample number
**Example**	TNBS: immersing larvae in 25–75 ug/mL TNBS from 3 dpf	OXO: 0.2% oxazolone by intrarectally injection
	DSS: immersing larvae 0.5% (*w*/*v*) DSS from 3dpf	TNBS: 1 uL per 0.1g of body weight by intrarectally injection

TNBS: 2,4,6-trinitrobenzene sulfonic acid; DSS: Dextran sodium sulfate; OXO: oxazolone.

## References

[1] Wong M.C., Ching J.Y., Chan V.C., Lam T.Y., Luk A.K., Wong S.H., Ng S.C., Ng S.S., Wu J.C., Chan F.K. (2016). Colorectal cancer screening based on age and gender: A cost-effectiveness analysis. Medicine.

[2] Markowitz S.D., Bertagnolli M.M. (2009). Molecular origins of cancer: Molecular basis of colorectal cancer. N. Eng. J. Med..

[3] Vogelstein B., Kinzler K.W. (2015). The path to cancer—Three strikes and you’re out. N. Engl. J. Med..

[4] Wang T.L., Rago C., Silliman N., Ptak J., Markowitz S., Willson J.K., Parmigiani G., Kinzler K.W., Vogelstein B., Velculescu V.E. (2002). Prevalence of somatic alterations in the colorectal cancer cell genome. Proc. Natl. Acad. Sci. USA.

[5] Mann B., Gelos M., Siedow A., Hanski M.L., Gratchev A., Ilyas M., Bodmer W.F., Moyer M.P., Riecken E.O., Buhr H.J. (1999). Target genes of beta-catenin-t cell-factor/lymphoid-enhancer-factor signaling in human colorectal carcinomas. Proc. Natl. Acad. Sci. USA.

[6] Tariq K., Ghias K. (2016). Colorectal cancer carcinogenesis: A review of mechanisms. Cancer Biol. Med..

[7] Christie M., Jorissen R.N., Mouradov D., Sakthianandeswaren A., Li S., Day F., Tsui C., Lipton L., Desai J., Jones I.T. (2013). Different apc genotypes in proximal and distal sporadic colorectal cancers suggest distinct wnt/beta-catenin signalling thresholds for tumourigenesis. Oncogene.

[8] Dow L.E., O’Rourke K.P., Simon J., Tschaharganeh D.F., van Es J.H., Clevers H., Lowe S.W. (2015). Apc restoration promotes cellular differentiation and reestablishes crypt homeostasis in colorectal cancer. Cell.

[9] Watanabe K., Biesinger J., Salmans M.L., Roberts B.S., Arthur W.T., Cleary M., Andersen B., Xie X., Dai X. (2014). Integrative chip-seq/microarray analysis identifies a ctnnb1 target signature enriched in intestinal stem cells and colon cancer. PLoS ONE.

[10] Vogelstein B., Fearon E.R., Hamilton S.R., Kern S.E., Preisinger A.C., Leppert M., Nakamura Y., White R., Smits A.M., Bos J.L. (1988). Genetic alterations during colorectal-tumor development. N. Engl. J. Med..

[11] Pretlow T.P., Pretlow T.G. (2005). Mutant kras in aberrant crypt foci (acf): Initiation of colorectal cancer?. Biochim. Biophys. Acta.

[12] Rosty C., Young J.P., Walsh M.D., Clendenning M., Walters R.J., Pearson S., Pavluk E., Nagler B., Pakenas D., Jass J.R. (2013). Colorectal carcinomas with kras mutation are associated with distinctive morphological and molecular features. Mod. Pathol..

[13] Li W., Qiu T., Zhi W., Shi S., Zou S., Ling Y., Shan L., Ying J., Lu N. (2015). Colorectal carcinomas with kras codon 12 mutation are associated with more advanced tumor stages. BMC Cancer.

[14] Pino M.S., Chung D.C. (2010). The chromosomal instability pathway in colon cancer. Gastroenterology.

[15] Kosmidou V., Oikonomou E., Vlassi M., Avlonitis S., Katseli A., Tsipras I., Mourtzoukou D., Kontogeorgos G., Zografos G., Pintzas A. (2014). Tumor heterogeneity revealed by kras, braf, and pik3ca pyrosequencing: Kras and pik3ca intratumor mutation profile differences and their therapeutic implications. Hum. Mutat..

[16] Johnson S.M., Gulhati P., Rampy B.A., Han Y., Rychahou P.G., Doan H.Q., Weiss H.L., Evers B.M. (2010). Novel expression patterns of pi3k/akt/mtor signaling pathway components in colorectal cancer. J. Am. Coll. Surg..

[17] Suman S., Kurisetty V., Das T.P., Vadodkar A., Ramos G., Lakshmanaswamy R., Damodaran C. (2014). Activation of akt signaling promotes epithelial-mesenchymal transition and tumor growth in colorectal cancer cells. Mol. Carcinog..

[18] Chin Y.R., Yuan X., Balk S.P., Toker A. (2014). Pten-deficient tumors depend on akt2 for maintenance and survival. Cancer Discov..

[19] Ye Q., Cai W., Zheng Y., Evers B.M., She Q.B. (2014). Erk and akt signaling cooperate to translationally regulate survivin expression for metastatic progression of colorectal cancer. Oncogene.

[20] Cooks T., Pateras I.S., Tarcic O., Solomon H., Schetter A.J., Wilder S., Lozano G., Pikarsky E., Forshew T., Rosenfeld N. (2013). Mutant p53 prolongs nf-kappab activation and promotes chronic inflammation and inflammation-associated colorectal cancer. Cancer Cell.

[21] Zheng T., Wang J., Zhao Y., Zhang C., Lin M., Wang X., Yu H., Liu L., Feng Z., Hu W. (2013). Spliced mdm2 isoforms promote mutant p53 accumulation and gain-of-function in tumorigenesis. Nat. Commun..

[22] Brighenti E., Calabrese C., Liguori G., Giannone F.A., Trere D., Montanaro L., Derenzini M. (2014). Interleukin 6 downregulates p53 expression and activity by stimulating ribosome biogenesis: A new pathway connecting inflammation to cancer. Oncogene.

[23] Aparo S., Goel S. (2012). Evolvement of the treatment paradigm for metastatic colon cancer. From chemotherapy to targeted therapy. Crit. Rev. Oncol. Hematol..

[24] Recondo G., Diaz-Canton E., de la Vega M., Greco M., Recondo G., Valsecchi M.E. (2014). Advances and new perspectives in the treatment of metastatic colon cancer. World J. Gastrointest. Oncol..

[25] O’Connell M.J., Campbell M.E., Goldberg R.M., Grothey A., Seitz J.F., Benedetti J.K., Andre T., Haller D.G., Sargent D.J. (2008). Survival following recurrence in stage ii and iii colon cancer: Findings from the accent data set. J. Clin. Oncol..

[26] Heath J.K. (2010). Transcriptional networks and signaling pathways that govern vertebrate intestinal development. Curr. Top. Dev. Biol..

[27] Sander V., Davidson A.J. (2014). Kidney injury and regeneration in zebrafish. Semin. Nephrol..

[28] Wang Z., Du J., Lam S.H., Mathavan S., Matsudaira P., Gong Z. (2010). Morphological and molecular evidence for functional organization along the rostrocaudal axis of the adult zebrafish intestine. BMC Genom..

[29] Wallace K.N., Pack M. (2003). Unique and conserved aspects of gut development in zebrafish. Dev. Biol..

[30] Crosnier C., Vargesson N., Gschmeissner S., Ariza-McNaughton L., Morrison A., Lewis J. (2005). Delta-notch signalling controls commitment to a secretory fate in the zebrafish intestine. Development.

[31] Ng A.N., de Jong-Curtain T.A., Mawdsley D.J., White S.J., Shin J., Appel B., Dong P.D., Stainier D.Y., Heath J.K. (2005). Formation of the digestive system in zebrafish: Iii. Intestinal epithelium morphogenesis. Dev. Biol..

[32] Wallace K.N., Akhter S., Smith E.M., Lorent K., Pack M. (2005). Intestinal growth and differentiation in zebrafish. Mech. Dev..

[33] Mudumana S.P., Wan H., Singh M., Korzh V., Gong Z. (2004). Expression analyses of zebrafish transferrin, ifabp, and elastaseb mrnas as differentiation markers for the three major endodermal organs: Liver, intestine, and exocrine pancreas. Dev. Dyn..

[34] Nalbant P., Boehmer C., Dehmelt L., Wehner F., Werner A. (1999). Functional characterization of a na^+^—Phosphate cotransporter (napi-ii) from zebrafish and identification of related transcripts. J. Physiol..

[35] Stuart G.W., McMurray J.V., Westerfield M. (1988). Replication, integration and stable germ-line transmission of foreign sequences injected into early zebrafish embryos. Development.

[36] Culp P., Nusslein-Volhard C., Hopkins N. (1991). High-frequency germ-line transmission of plasmid DNA sequences injected into fertilized zebrafish eggs. Proc. Natl. Acad. Sci. USA.

[37] Lu J.W., Ho Y.J., Yang Y.J., Liao H.A., Ciou S.C., Lin L.I., Ou D.L. (2015). Zebrafish as a disease model for studying human hepatocellular carcinoma. World J. Gastroenterol..

[38] Park S.W., Davison J.M., Rhee J., Hruban R.H., Maitra A., Leach S.D. (2008). Oncogenic kras induces progenitor cell expansion and malignant transformation in zebrafish exocrine pancreas. Gastroenterology.

[39] Stuart G.W., Vielkind J.R., McMurray J.V., Westerfield M. (1990). Stable lines of transgenic zebrafish exhibit reproducible patterns of transgene expression. Development.

[40] Thermes V., Grabher C., Ristoratore F., Bourrat F., Choulika A., Wittbrodt J., Joly J.S. (2002). I-scei meganuclease mediates highly efficient transgenesis in fish. Mech. Dev..

[41] Ivics Z., Hackett P.B., Plasterk R.H., Izsvak Z. (1997). Molecular reconstruction of sleeping beauty, a tc1-like transposon from fish, and its transposition in human cells. Cell.

[42] Emelyanov A., Gao Y., Naqvi N.I., Parinov S. (2006). Trans-kingdom transposition of the maize dissociation element. Genetics.

[43] Kawakami K., Shima A. (1999). Identification of the tol2 transposase of the medaka fish oryzias latipes that catalyzes excision of a nonautonomous tol2 element in zebrafish danio rerio. Gene.

[44] Izsvak Z., Ivics Z., Hackett P.B. (1995). Characterization of a tc1-like transposable element in zebrafish (danio rerio). Mol. Gen. Genet..

[45] Ivics Z., Izsvak Z., Minter A., Hackett P.B. (1996). Identification of functional domains and evolution of tc1-like transposable elements. Proc. Natl. Acad. Sci. USA.

[46] Davidson A.E., Balciunas D., Mohn D., Shaffer J., Hermanson S., Sivasubbu S., Cliff M.P., Hackett P.B., Ekker S.C. (2003). Efficient gene delivery and gene expression in zebrafish using the sleeping beauty transposon. Dev. Biol..

[47] Huang X., Nguyen A.T., Li Z., Emelyanov A., Parinov S., Gong Z. (2011). One step forward: The use of transgenic zebrafish tumor model in drug screens. Birth Defects Res. C Embryo Today.

[48] Deiters A., Yoder J.A. (2006). Conditional transgene and gene targeting methodologies in zebrafish. Zebrafish.

[49] Her G.M., Chiang C.C., Wu J.L. (2004). Zebrafish intestinal fatty acid binding protein (i-fabp) gene promoter drives gut-specific expression in stable transgenic fish. Genesis.

[50] Her G.M., Yeh Y.H., Wu J.L. (2004). Functional conserved elements mediate intestinal-type fatty acid binding protein (i-fabp) expression in the gut epithelia of zebrafish larvae. Dev. Dyn..

[51] Neal J.T., Peterson T.S., Kent M.L., Guillemin K.H. (2013). Pylori virulence factor caga increases intestinal cell proliferation by wnt pathway activation in a transgenic zebrafish model. Dis. Model. Mech..

[52] Le X., Langenau D.M., Keefe M.D., Kutok J.L., Neuberg D.S., Zon L.I. (2007). Heat shock-inducible cre/lox approaches to induce diverse types of tumors and hyperplasia in transgenic zebrafish. Proc. Natl. Acad. Sci. USA.

[53] Dooley K., Zon L.I. (2000). Zebrafish: A model system for the study of human disease. Curr. Opin. Genet. Dev..

[54] Walter R.B., Kazianis S. (2001). Xiphophorus interspecies hybrids as genetic models of induced neoplasia. ILAR J..

[55] Spitsbergen J.M., Tsai H.W., Reddy A., Miller T., Arbogast D., Hendricks J.D., Bailey G.S. (2000). Neoplasia in zebrafish (danio rerio) treated with 7,12-dimethylbenz[a]anthracene by two exposure routes at different developmental stages. Toxicol. Pathol..

[56] Amatruda J.F., Shepard J.L., Stern H.M., Zon L.I. (2002). Zebrafish as a cancer model system. Cancer Cell.

[57] Stern H.M., Zon L.I. (2003). Cancer genetics and drug discovery in the zebrafish. Nat. Rev. Cancer.

[58] Paquette C.E., Kent M.L., Buchner C., Tanguay R.L., Guillemin K., Mason T.J., Peterson T.S. (2013). A retrospective study of the prevalence and classification of intestinal neoplasia in zebrafish (danio rerio). Zebrafish.

[59] Paquette C.E., Kent M.L., Peterson T.S., Wang R., Dashwood R.H., Lohr C.V. (2015). Immunohistochemical characterization of intestinal neoplasia in zebrafish danio rerio indicates epithelial origin. Dis. Aquat. Organ..

[60] Groden J., Thliveris A., Samowitz W., Carlson M., Gelbert L., Albertsen H., Joslyn G., Stevens J., Spirio L., Robertson M. (1991). Identification and characterization of the familial adenomatous polyposis coli gene. Cell.

[61] Kinzler K.W., Nilbert M.C., Su L.K., Vogelstein B., Bryan T.M., Levy D.B., Smith K.J., Preisinger A.C., Hedge P., McKechnie D. (1991). Identification of fap locus genes from chromosome 5q21. Science.

[62] Kinzler K.W., Vogelstein B. (1996). Lessons from hereditary colorectal cancer. Cell.

[63] Su L.K., Kinzler K.W., Vogelstein B., Preisinger A.C., Moser A.R., Luongo C., Gould K.A., Dove W.F. (1992). Multiple intestinal neoplasia caused by a mutation in the murine homolog of the apc gene. Science.

[64] Bienz M., Clevers H. (2000). Linking colorectal cancer to wnt signaling. Cell.

[65] Hurlstone A.F., Haramis A.P., Wienholds E., Begthel H., Korving J., Van Eeden F., Cuppen E., Zivkovic D., Plasterk R.H., Clevers H. (2003). The wnt/beta-catenin pathway regulates cardiac valve formation. Nature.

[66] Haramis A.P., Hurlstone A., van der Velden Y., Begthel H., van den Born M., Offerhaus G.J., Clevers H.C. (2006). Adenomatous polyposis coli-deficient zebrafish are susceptible to digestive tract neoplasia. EMBO Rep..

[67] Sandoval I.T., Delacruz R.G., Miller B.N., Hill S., Olson K.A., Gabriel A.E., Boyd K., Satterfield C., Remmen H.V., Rutter J. (2017). A metabolic switch controls intestinal differentiation downstream of adenomatous polyposis coli (apc). Elife.

[68] Burger A., Vasilyev A., Tomar R., Selig M.K., Nielsen G.P., Peterson R.T., Drummond I.A., Haber D.A. (2014). A zebrafish model of chordoma initiated by notochord-driven expression of hrasv12. Dis. Model. Mech..

[69] Santoriello C., Gennaro E., Anelli V., Distel M., Kelly A., Koster R.W., Hurlstone A., Mione M. (2010). Kita driven expression of oncogenic hras leads to early onset and highly penetrant melanoma in zebrafish. PLoS ONE.

[70] Storer N.Y., White R.M., Uong A., Price E., Nielsen G.P., Langenau D.M., Zon L.I. (2013). Zebrafish rhabdomyosarcoma reflects the developmental stage of oncogene expression during myogenesis. Development.

[71] Dovey M., White R.M., Zon L.I. (2009). Oncogenic nras cooperates with p53 loss to generate melanoma in zebrafish. Zebrafish.

[72] Ju B., Chen W., Orr B.A., Spitsbergen J.M., Jia S., Eden C.J., Henson H.E., Taylor M.R. (2015). Oncogenic kras promotes malignant brain tumors in zebrafish. Mol. Cancer.

[73] Shive H.R., West R.R., Embree L.J., Sexton J.M., Hickstein D.D. (2015). Expression of krasg12v in zebrafish gills induces hyperplasia and cxcl8-associated inflammation. Zebrafish.

[74] Nguyen A.T., Emelyanov A., Koh C.H., Spitsbergen J.M., Lam S.H., Mathavan S., Parinov S., Gong Z. (2011). A high level of liver-specific expression of oncogenic kras(v12) drives robust liver tumorigenesis in transgenic zebrafish. Dis. Model. Mech..

[75] Provost E., Bailey J.M., Aldrugh S., Liu S., Iacobuzio-Donahue C., Leach S.D. (2014). The tumor suppressor rpl36 restrains kras(g12v)-induced pancreatic cancer. Zebrafish.

[76] Bos J.L., Fearon E.R., Hamilton S.R., Verlaan-de Vries M., van Boom J.H., van der Eb A.J., Vogelstein B. (1987). Prevalence of ras gene mutations in human colorectal cancers. Nature.

[77] Berghmans S., Murphey R.D., Wienholds E., Neuberg D., Kutok J.L., Fletcher C.D., Morris J.P., Liu T.X., Schulte-Merker S., Kanki J.P. (2005). Tp53 mutant zebrafish develop malignant peripheral nerve sheath tumors. Proc. Natl. Acad. Sci. USA.

[78] Lu J.W., Yang W.Y., Tsai S.M., Lin Y.M., Chang P.H., Chen J.R., Wang H.D., Wu J.L., Jin S.L., Yuh C.H. (2013). Liver-specific expressions of hbx and src in the p53 mutant trigger hepatocarcinogenesis in zebrafish. PLoS ONE.

[79] Faro A., Boj S.F., Clevers H. (2009). Fishing for intestinal cancer models: Unraveling gastrointestinal homeostasis and tumorigenesis in zebrafish. Zebrafish.

[80] Barton C.M., Staddon S.L., Hughes C.M., Hall P.A., O’Sullivan C., Kloppel G., Theis B., Russell R.C., Neoptolemos J., Williamson R.C. (1991). Abnormalities of the p53 tumour suppressor gene in human pancreatic cancer. Br. J. Cancer.

[81] Hsu I.C., Metcalf R.A., Sun T., Welsh J.A., Wang N.J., Harris C.C. (1991). Mutational hotspot in the p53 gene in human hepatocellular carcinomas. Nature.

[82] Baker S.J., Fearon E.R., Nigro J.M., Hamilton S.R., Preisinger A.C., Jessup J.M., vanTuinen P., Ledbetter D.H., Barker D.F., Nakamura Y. (1989). Chromosome 17 deletions and p53 gene mutations in colorectal carcinomas. Science.

[83] Zon L.I., Peterson R.T. (2005). In vivo drug discovery in the zebrafish. Nat. Rev. Drug Discov..

[84] Tamplin O.J., White R.M., Jing L., Kaufman C.K., Lacadie S.A., Li P., Taylor A.M., Zon L.I. (2012). Small molecule screening in zebrafish: Swimming in potential drug therapies. Wiley Interdiscip. Rev. Dev. Biol..

[85] Peterson R.T., Macrae C.A. (2012). Systematic approaches to toxicology in the zebrafish. Annu. Rev. Pharmacol. Toxicol..

[86] Cariati M., Marlow R., Dontu G. (2011). Xenotransplantation of breast cancers. Methods Mol. Biol..

[87] White R.M., Sessa A., Burke C., Bowman T., LeBlanc J., Ceol C., Bourque C., Dovey M., Goessling W., Burns C.E. (2008). Transparent adult zebrafish as a tool for in vivo transplantation analysis. Cell Stem Cell.

[88] Lawson N.D., Weinstein B.M. (2002). In vivo imaging of embryonic vascular development using transgenic zebrafish. Dev. Biol..

[89] Konantz M., Balci T.B., Hartwig U.F., Dellaire G., Andre M.C., Berman J.N., Lengerke C. (2012). Zebrafish xenografts as a tool for in vivo studies on human cancer. Ann. N. Y. Acad. Sci..

[90] Tang Q., Abdelfattah N.S., Blackburn J.S., Moore J.C., Martinez S.A., Moore F.E., Lobbardi R., Tenente I.M., Ignatius M.S., Berman J.N. (2014). Optimized cell transplantation using adult rag2 mutant zebrafish. Nat. Methods.

[91] Tang Q., Moore J.C., Ignatius M.S., Tenente I.M., Hayes M.N., Garcia E.G., Torres Yordan N., Bourque C., He S., Blackburn J.S. (2016). Imaging tumour cell heterogeneity following cell transplantation into optically clear immune-deficient zebrafish. Nat. Commun..

[92] Veinotte C.J., Dellaire G., Berman J.N. (2014). Hooking the big one: The potential of zebrafish xenotransplantation to reform cancer drug screening in the genomic era. Dis. Model. Mech..

[93] Haldi M., Ton C., Seng W.L., McGrath P. (2006). Human melanoma cells transplanted into zebrafish proliferate, migrate, produce melanin, form masses and stimulate angiogenesis in zebrafish. Angiogenesis.

[94] Gnosa S., Capodanno A., Murthy R.V., Jensen L.D., Sun X.F. (2016). Aeg-1 knockdown in colon cancer cell lines inhibits radiation-enhanced migration and invasion in vitro and in a novel in vivo zebrafish model. Oncotarget.

[95] Roel M., Rubiolo J.A., Guerra-Varela J., Silva S.B., Thomas O.P., Cabezas-Sainz P., Sanchez L., Lopez R., Botana L.M. (2016). Marine guanidine alkaloids crambescidins inhibit tumor growth and activate intrinsic apoptotic signaling inducing tumor regression in a colorectal carcinoma zebrafish xenograft model. Oncotarget.

[96] Fior R., Povoa V., Mendes R.V., Carvalho T., Gomes A., Figueiredo N., Ferreira M.G. (2017). Single-cell functional and chemosensitive profiling of combinatorial colorectal therapy in zebrafish xenografts. Proc. Natl. Acad. Sci. USA.

[97] Dang M., Henderson R.E., Garraway L.A., Zon L.I. (2016). Long-term drug administration in the adult zebrafish using oral gavage for cancer preclinical studies. Dis. Model. Mech..

[98] Brugman S. (2016). The zebrafish as a model to study intestinal inflammation. Dev. Comp. Immunol..

[99] Brugman S., Liu K.Y., Lindenbergh-Kortleve D., Samsom J.N., Furuta G.T., Renshaw S.A., Willemsen R., Nieuwenhuis E.E. (2009). Oxazolone-induced enterocolitis in zebrafish depends on the composition of the intestinal microbiota. Gastroenterology.

[100] Fleming A., Jankowski J., Goldsmith P. (2010). In vivo analysis of gut function and disease changes in a zebrafish larvae model of inflammatory bowel disease: A feasibility study. Inflamm. Bowel Dis..

[101] Oehlers S.H., Flores M.V., Okuda K.S., Hall C.J., Crosier K.E., Crosier P.S. (2011). A chemical enterocolitis model in zebrafish larvae that is dependent on microbiota and responsive to pharmacological agents. Dev. Dyn..

[102] Geiger B.M., Gras-Miralles B., Ziogas D.C., Karagiannis A.K., Zhen A., Fraenkel P., Kokkotou E. (2013). Intestinal upregulation of melanin-concentrating hormone in tnbs-induced enterocolitis in adult zebrafish. PLoS ONE.

[103] He Q., Wang L., Wang F., Wang C., Tang C., Li Q., Li J., Zhao Q. (2013). Microbial fingerprinting detects intestinal microbiota dysbiosis in zebrafish models with chemically-induced enterocolitis. BMC Microbiol..

[104] He Q., Wang L., Wang F., Li Q. (2014). Role of gut microbiota in a zebrafish model with chemically induced enterocolitis involving toll-like receptor signaling pathways. Zebrafish.

[105] Wirtz S., Neufert C., Weigmann B., Neurath M.F. (2007). Chemically induced mouse models of intestinal inflammation. Nat. Protoc..

[106] Oehlers S.H., Flores M.V., Hall C.J., Crosier K.E., Crosier P.S. (2012). Retinoic acid suppresses intestinal mucus production and exacerbates experimental enterocolitis. Dis. Model. Mech..

[107] Oehlers S.H., Flores M.V., Hall C.J., Okuda K.S., Sison J.O., Crosier K.E., Crosier P.S. (2013). Chemically induced intestinal damage models in zebrafish larvae. Zebrafish.

[108] Oehlers S.H., Flores M.V., Hall C.J., Wang L., Ko D.C., Crosier K.E., Crosier P.S. (2017). A whole animal chemical screen approach to identify modifiers of intestinal neutrophilic inflammation. FEBS J..

[109] Okuda K.S., Misa J.P., Oehlers S.H., Hall C.J., Ellett F., Alasmari S., Lieschke G.J., Crosier K.E., Crosier P.S., Astin J.W. (2015). A zebrafish model of inflammatory lymphangiogenesis. Biol. Open.

